# Analytical Methods for Melatonin Quantification: Advances, Challenges, and Clinical Applications

**DOI:** 10.3390/ph19030439

**Published:** 2026-03-09

**Authors:** Mihaela Butiulca, Lenard Farczadi, Mihaly Veres, Leonard Azamfirei

**Affiliations:** 1 Department of Anesthesiology and Intensive Care Medicine, Faculty of General Medicine, George Emil Palade University of Medicine, Pharmacy, Science, and Technology of Targu Mures, 540142 Targu Mures, Romania; mihaela.budrescu@umfst.ro (M.B.);; 2 Department of Anesthesiology and Intensive Care Medicine, Emergency County Hospital, 540142 Targu Mures, Romania; 3 Chromatography and Mass Spectrometry Laboratory, Centre for Advanced Medical and Pharmaceutical Research, George Emil Palade University of Medicine, Pharmacy, Science, and Technology of Targu Mures, 540142 Targu Mures, Romania; 4 Doctoral School of Medicine and Pharmacy, George Emil Palade University of Medicine, Pharmacy, Science, and Technology of Targu Mures, 540142 Targu Mures, Romania

**Keywords:** melatonin, analytical techniques, LC–MS/MS, circadian biomarkers, pharmacology

## Abstract

Melatonin, an indoleamine crucial for regulating circadian rhythms, sleep–wake cycles, and immune–endocrine homeostasis, is present in biological fluids at extremely low concentrations, making its quantification analytically challenging. This narrative review provides a critical comparative assessment of current methodologies for melatonin determination across various biological matrices—plasma, urine, saliva, breast milk, and hair. The discussed techniques include immunoassays, colorimetric and spectrophotometric methods, chromatographic–mass spectrometric platforms (LC–MS/MS, UHPLC–MS/MS), and emerging biosensors. Each approach is evaluated regarding analytical sensitivity, specificity, reproducibility, cost, and clinical applicability. While immunoenzymatic and colorimetric techniques offer accessible, low-cost solutions for large-scale or preliminary studies, LC–MS/MS remains the benchmark for reference analysis, providing sub-picogram detection limits and multiplexing capability. However, its high cost, procedural complexity, and inter-laboratory variability limit routine implementation. New developments, including molecularly imprinted polymers, dispersive microextraction, and nanomaterial-based biosensors, suggest a shift toward hybrid, sustainable, and portable analytical platforms. By synthesizing recent methodological advances and identifying key limitations, this review aims to guide researchers and clinicians in selecting the most appropriate analytical approach for clinical, pharmacological, and circadian biomonitoring applications.

## 1. Introduction

**Figure 1:**
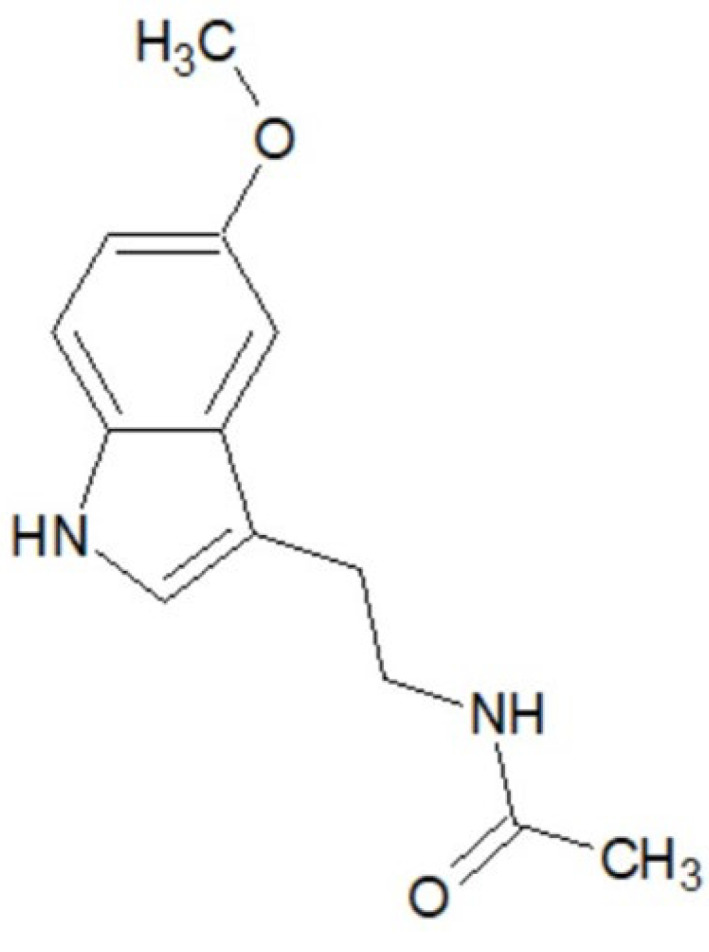
Chemical structure of melatonin.

Melatonin (N-acetyl-5-methoxytryptamine) is an endogenous indoleamine hormone that plays a crucial role in regulating circadian rhythms, sleep–wake cycles, immune modulation, and seasonal endocrine function. Accurate quantification of melatonin is essential not only in neuroendocrinological studies but also in clinical research on sleep disorders, depression, and delirium [1,2]. Structurally, melatonin derives from the essential amino acid tryptophan. It contains an indole nucleus substituted at the 5-position with a methoxy group (–OCH_3_), while the ethyl side chain at the 3-position carries an acetamide group (–NHCOCH_3_). Its physicochemical profile—aromatic stability coupled with moderate lipophilicity—facilitates rapid diffusion through biological membranes. This explains the hormone’s wide distribution and its presence across diverse biological matrices such as plasma, saliva, urine, breast milk, and even hair (Figure 1) [3].

Because physiological concentrations are typically below 10 pg/mL during the day, melatonin quantification represents a significant analytical challenge. Over time, several quantification strategies have been developed, including immunoassays (e.g., ELISA, RIA), spectrophotometric techniques, and mass spectrometry (LC–MS/MS, GC–MS). Each technique varies in analytical sensitivity, specificity, cost, and operational complexity [1,4].

While immunoassays remain widely available and easy to use, they often suffer from cross-reactivity and limited specificity, particularly when applied to saliva samples or low-concentration matrices. In contrast, chromatographic–mass spectrometric methods are known to be more sensible and specific in quantification of various substances. However, these high-precision methods require costly instrumentation, trained personnel, and stringent validation [1,2,5].

All aspects considered, however, given the ever increasing importance of metabolomics, generally, and melatonin quantification, specifically, it is important to have a wide range of reliable and cost-efficient methodologies available, which can be adapted and used in various fields of interest and in a wide range of clinical and pharmacological applications, and by as wide of a range of clinical and research experts as possible. In this regard, melatonin quantification is essential because it provides a precise biochemical marker of endogenous circadian phase and pineal gland function, enabling rigorous assessment of sleep–wake regulation, chronobiological disorders, and the physiological impact of therapeutic or environmental interventions. Melatonin quantification also supports sleep disorder diagnostics (e.g., DLMO), chronopharmacology, and ICU delirium prevention [2]. Our multi-platform focus fills gaps in prior narrow-scope reviews. A wide array of methods with various advantages and disadvantages are essential to cover all areas of clinical and scientific quantification, also with consideration to time and costs; however, as affirmed by Monga et al. (2024), LC–MS/MS remains to be considered the gold standard for sensitivity in sleep disorder biomarkers as well as other metabolomics applications [6].

Given these challenges, a structured and critical comparison of available techniques is essential to guide researchers and clinicians in selecting appropriate quantification tools. This review aims to summarize the recent advances in melatonin quantification. By evaluating the strengths and weaknesses of each analytical platform, we propose practical recommendations for their use in clinical, pharmacological, and translational contexts.

## 2. Methods

This article was designed as a narrative review. The objective was to critically assess the main analytical techniques currently applied for melatonin quantification in human biological matrices. We also aim to evaluate their technical performance and clinical applicability. A comprehensive literature search was carried out between July and December 2025. Three academic databases—PubMed, Web of Science, and Scopus—were queried using combinations of the following keywords: “melatonin quantification”, “LC–MS/MS detection AND melatonin”, and “melatonin biomonitoring”. The search was restricted to peer-reviewed articles published between January 2020 and September 2025, with a focus on the last five years to ensure up-to-date methodological relevance. From a total of 248 hits, a total of 217 manuscripts were excluded due to being duplicates, discussed in other manuscripts extensively or being irrelevant (for example, 40 manuscripts focusing on non-human research), as well as 150 manuscripts being filtered out due to being published prior to 2020, and a total of 31 manuscript were included in this review.

### Novelty of the Study

This review provides a unified technical perspective on melatonin quantification by integrating evidence from immunoassays, spectrophotometric methods, chromatography–mass spectrometry, and emerging biosensors. It brings novelty by comparing these platforms across multiple biological matrices and by emphasizing how pre-analytical variables affect analytical performance. The work synthesizes recent advances in microextraction, molecularly imprinted materials, and nanostructured sensing technologies. It shows how these tools can bridge the gap between low-cost screening and high-precision quantification. It also highlights the growing trend toward multiplex biomarker profiling, where melatonin is measured alongside cortisol, cortisone, or serotonin metabolites. The review examines the methodological trade-offs in light of current regulatory requirements and available inter-laboratory harmonization data. A structured framework for choosing the most suitable method can be achieved by connecting analytical limitations with practical needs in clinical and translational research. This multidimensional perspective sets the review apart from earlier publications that tend to focus on only one analytical approach.

Unlike prior reviews (e.g., Kennaway 2019 [1], Monga 2024 [6]), this work uniquely integrates MIP-microextraction ([7]) and biosensor advances ([8]) with salivary aMT6s reliability for immunoassays.

## 3. Analytical Techniques Overview

Methods for measuring melatonin have changed considerably over the past decades, in line with the increasing interest in this hormone and its metabolites. Analytical methods differ widely in terms of sensitivity, specificity, cost, processing time, and applicability to various biological matrices [1].

### 3.1. Spectrophotometric and Colorimetric Techniques

Recent innovations have aimed to develop low-cost and portable melatonin assays compatible with point-of-care (POC) testing. Khachornsakkul et al. (2023) [9] proposes the use of distance-based thread analytical devices (dTADs) combined with dispersive liquid–liquid microextraction (DLLME). This colorimetric method allows melatonin detection in saliva, urine, and serum without relying on advanced instruments. The procedure extracts melatonin into chloroform and then reacts it with 2,3-naphthalenedialdehyde (2,3-Nda) fixed onto a cotton thread, which produces a yellow–orange band. The length of this colored band reflects the melatonin concentration, providing a simple and low-cost way to estimate its levels. The technique has demonstrated high analytical performance, with linearity in the range of 15.0–45.0 pg/mL (R^2^ = 0.9949), a low detection limit of 2.50 pg/mL, and recovery rates between 98.42% and 102.52%. Despite its portability and low cost, the reliance on visual signal length introduces subjectivity. It highlights the need for digital enhancement and further standardization [9].

Another technique using dispersive solid-phase microextraction (DSPME) was described by Dil et al. [10]. They combined magnetic nanoparticles with UV–Vis (Ultraviolet-visible) spectrophotometry for selective melatonin quantification in serum and urine. Here, Fe_3_O_4_@SiO_2_ nanoparticles coated with a melatonin-specific molecularly imprinted polymer (MIP) selectively extract the analyte. The analyte is then eluted and quantified by absorbance at 278 nm. This method showed a wide linear range (2–500 ng/mL), low detection limits (0.67 ng/mL), and high enrichment (factor of 62). The precision (RSD < 4.8%) and recovery (93.6–102.2%) comparable to reference methods. While this technique aligns with principles of green chemistry—due to minimal solvent use and fast processing—its dependence on UV–Vis detection introduces vulnerability to spectral interferences. This limits its utility in complex matrices without additional validation [10].

### 3.2. Chromatography–Mass Spectrometry (LC–MS/MS and UHPLC–MS/MS)

Martinez-Moral and Kannan (2025) [11] developed a UPLC–MS/MS (Ultra-Performance Liquid Chromatography coupled with Tandem Mass Spectrometry) method using low-density solvent DLLME for simultaneous determination of multiple substances, such as melatonin, cortisol, and 12 urinary metabolites. The protocol includes enzymatic deconjugation and MTBE (Methyl tert-butyl ether) extraction, achieving detection limits as low as 0.013 ng/mL and a linear range spanning over four orders of magnitude (0.02–750 ng/mL). Using isotopically labeled internal standards helps keep the measurements reliable. The multiplex setup also makes it easier to examine several circadian or stress-related biomarkers at the same time. Multiplexing capability refers to the ability of an analytical method to measure multiple analytes simultaneously within the same sample and in a single analytical run. The method still relies on enzymatic hydrolysis and several extraction steps, which makes routine use more difficult. Further method development has focused on improving matrix specificity and environmental compatibility. Machado et al. (2025) [7] introduced an LC–MS/MS workflow using MIP–DPX (Molecularly Imprinted Polymer—Disposable Pipette Extraction) extraction for melatonin detection in human breast milk. The use of MIP significantly minimized matrix interference, crucial in lipid-rich samples. The method achieved limits of detection down to 0.01 ng/mL and met EMA/FDA (European Medicines Agency/U.S. Food and Drug Administration) guidelines for reproducibility. Clinically, this approach provides insight into maternal–infant circadian synchronization via melatonin transmission through milk—an angle not addressed by urinary-based protocols like that of Martinez-Moral and Kannan [7,11].

Other researchers have optimized LC–MS/MS techniques across diverse sample types and research contexts. Yokokawa et al. (2024) [12] developed a method for simultaneous analysis of melatonin, caffeine, and paraxanthine in plasma with sub-picogram sensitivity (LLOQ (Lower Limit of Quantification) 0.487 pg/mL). Their work showed why isotopic internal standards are essential when dealing with analytes that have different endogenous levels. Magliocco et al. (2021) [13] later introduced a simpler urinary assay that avoids enzymatic hydrolysis and focuses only on the unconjugated melatonin metabolites. This strategy made the procedure faster and less complicated. It also narrowed the range of metabolites that could be detected and may have missed important conjugated forms. In parallel, other groups expanded the use of LC–MS/MS to retrospective and non-invasive sample types. Zhu et al. (2022) [14] applied this method to scalp hair, enabling month-scale hormonal profiling. Eugster et al. (2022) [15] and Kaleta et al. (2025) [16] expanded the scope by simultaneously quantifying serotonin and melatonin pathway metabolites. They are offering a more comprehensive view of tryptophan metabolism and its implications in circadian rhythm [12,13,14,15,16].

Xu et al. (2023) [17] implemented a UPLC–MS/MS method to monitor melatonin, 6-hydroxymelatonin sulfate, cortisol, cortisone, and 5-HIAA in air traffic controllers, demonstrating high sensitivity (LOD 0.05–1 ng/mL), precision (RSD < 8.3%), and reproducibility in occupational health settings. These studies reflect the evolution toward comprehensive metabolic profiling, where melatonin is contextualized within broader neuroendocrine pathways rather than measured in isolation [17]. Another study by Kaleta et al. (2025) [16] developed a fast and sensitive UHPLC–MS/MS method for the simultaneous quantification of seven tryptophan–melatonin pathway metabolites in human plasma [15].

Despite their analytical performance, MS-based platforms are expensive, require trained operators, and are prone to carry-over contamination when analyzing trace-level compounds. Consistent results require careful cleaning procedures, the use of internal standards, and regular calibration across laboratories [18].

### 3.3. Emerging Biosensor Technologies

Recent progress in nanotechnology has enabled the creation of optical and electrochemical biosensors capable of detecting melatonin at picomolar levels in real time. Duhan et al. [8] reviewed recent innovations incorporating nanostructured materials—such as graphene, gold nanoparticles, and quantum dots—into highly sensitive optical biosensors. These platforms detect melatonin through fluorescence, chemiluminescence, or plasmonic absorption, allowing for rapid, non-invasive, and reusable assays with detection limits in the picomolar range. These systems could be adapted for point-of-care devices and even wearable formats, allowing real-time hormone monitoring in sleep medicine, chronotherapy, and personalized care [8]. Sofu et al. (2025) [19] describe the development of a low-cost electrochemical sensor for detecting melatonin in biological and pharmaceutical samples. The device is based on a carbon paste electrode modified with a polymer layer, a design that improves both sensitivity and selectivity without requiring complex or expensive instrumentation. Because the sensor is simple to prepare and inexpensive to operate, it offers a practical alternative for melatonin analysis in laboratories that do not have access to high-end platforms such as LC–MS/MS [19].

Still, several issues need to be resolved, including signal standardization, variability across biological matrices, and the lack of large clinical validation studies. LOD/linear ranges degrade in matrices, with LOD values in reported being <1 pM for buffers compared to 5 pM for saliva, and much higher linearity ranges of 0.01–10 nM for saliva [8]. Antifouling via PEG/graphene (80% reduction in adsorption); calibration uses spiked standards or smartphone apps. Clinical validation of biosensors developed by Sofu et al. (LOD 24 nM plasma) correlates well with LC–MS/MS (r = 0.92, *n* = 50) but underperforms ELISA in low-range urine [19].

### 3.4. Immunoassay-Based Techniques

Immunoassays such as enzyme-linked immunosorbent assays (ELISA) and radioimmunoassay (RIA) remain widely employed due to their accessibility, affordability, and operational simplicity. However, they are prone to cross-reactivity and poor specificity, especially in saliva samples where melatonin concentrations are very low. Oliveira et al. (2022) [20] applied a commercial ELISA kit to quantify melatonin concentrations in a controlled in vitro study evaluating osteoblast activity in response to melatonin-enriched collagen membranes. Although the authors did not report a detection limit or linear range, the method allowed reliable tracking of melatonin levels in the culture media under different experimental conditions. Because it is simple and works well in uncomplicated matrices, ELISA remains useful for exploratory or mechanistic studies, especially when relative changes are more important than absolute values. However, the absence of internal standards and the potential for matrix effects underline the importance of confirmatory techniques in translational settings [20].

A systematic review by Rzepka-Migut and Paprocka [5] supports these conclusions, offering a detailed comparison of RIA, ELISA, HPLC, and LC–MS/MS methods across different biological matrices. The authors emphasize the impact of pre-analytical variables—such as light exposure and processing delays—on melatonin stability, particularly in saliva and plasma. While LC–MS/MS is endorsed as the reference standard, its limitations (e.g., cost, carry-over, and contamination risk) are acknowledged. The review highlights that rigorous methods are needed not only during detection but also in every step of sample handling. With this focus, it offers practical guidance for researchers who want to improve and standardize circadian biomonitoring protocols [5].

### 3.5. Comparative Summary of Analytical Methods

The comparison in Table 1 shows how different the available methods for measuring melatonin can be in terms of sensitivity, cost, processing time, and practical use. LC–MS/MS and UHPLC–MS/MS continue to be the most sensitive and dependable techniques, with detection limits below 1 pg/mL and consistently strong analytical performance. These strengths make them the standard choice for multiplex workflows that include cortisol, cortisone, or serotonin metabolites. Their drawbacks are mostly practical: high costs, longer processing times, and the need for trained staff. Colorimetric and spectrophotometric approaches, such as dTADs or DSPME–UV–Vis, are easier to implement and much faster, but they offer lower selectivity and are more vulnerable to matrix effects. ELISA provides a middle ground for large datasets or early-stage studies, although differences between kits and sample types can complicate comparisons. Table 1 also highlights a clear move toward smaller and more portable systems, especially among biosensor platforms, which may allow real-time measurements in point-of-care settings. Overall, no single method offers universal superiority—selection must be tailored to the intended biological matrix, study objectives, and logistical constraints [21].

## 4. Pre-Analytical Variability and Sample Handling

Pre-analytical factors are just as important as analytical accuracy when measuring melatonin. The molecule is quite unstable, and its levels can change quickly with light exposure, temperature, the type of sample, or delays in processing. Even short contact with normal room light can reduce the measurable concentration, especially in saliva and plasma. Its breakdown under ultraviolet and visible light is well documented, with losses of 20–40% occurring within minutes. To minimize this effect, samples must be collected and processed under dim or red-light conditions and stored immediately on ice or at –20 °C. The biological matrix also strongly influences stability. Urine samples and frozen plasma tend to remain stable for long periods, while saliva breaks down much faster if kept at room temperature [21,22]. Studies by Gröschl et al. (2008) [23] and Skubic et al. (2025) [24] showed that delays in centrifugation or freezing can change measured melatonin levels by as much as 50%, making comparisons between studies unreliable. The time between collection and freezing is also important and should ideally be under 30 min. Repeated freeze–thaw cycles should be avoided. The type of collection device can matter as well, since cotton and synthetic swabs differ in recovery and background interference. To keep results consistent, researchers need to standardize key aspects of sampling, such as the timing relative to the circadian cycle, the amount of light during collection, storage temperature, processing delays, and the choice of matrix and collection device. Pre-analytical standardization is therefore as important as analytical precision itself, serving as a prerequisite for reliable quantification regardless of the chosen detection technique. Pre-analytical variables are an essential part of the analytical process, and their study and clear definition ensures reliable and reproducible results for an analytical technique [5,20,24].

## 5. Discussion and Comparative Evaluation

Accurate quantification of melatonin depends on both analytical performance and pre-analytical control. Selecting an analytical method means weighing sensitivity, specificity, cost, and practical feasibility, depending on whether the aim is clinical diagnosis, pharmacokinetic work, or population-level monitoring [6].

### 5.1. Immunoassay Techniques: Utility and Limitations

Immunoassay-based methods such as ELISA and RIA are frequently used due to their low cost and operational simplicity. They are useful in large epidemiological studies, exploratory work, or in vitro experiments where relative changes are usually sufficient. Their main drawbacks are cross-reactivity, poor reproducibility at low concentrations, and variability between different production lots of commercial kits. For example, the IBL Saliva Melatonin ELISA kit has been reported to overestimate nocturnal levels compared to LC–MS/MS results, yielding only moderate correlation (r ≈ 0.65). Their technical limitations come mainly from the antibodies themselves. Cross-reactivity with indole derivatives and structurally related metabolites can inflate results. Sensitivity drops sharply at low concentrations, where the signal can be hard to separate from background noise. Batch-to-batch differences between kits may also change the calibration curve and shift results, even when the protocol is followed correctly. For this reason, consistent use requires strict internal controls and periodic checks against an external reference. Cross-validation with LC–MS/MS can help, and regression-based correction models often improve agreement in the mid-range of concentrations. Even so, ELISA is still not suitable for clinical trials that require sub-picogram precision [25].

For salivary melatonin, immunoassays often overestimate vs. LC–MS/MS [20], but excel for urinary 6-sulfatoxymelatonin (aMT6s), the principal metabolite (95% excreted form), where creatinine normalization enables robust circadian profiling without light sensitivity issues [20].

### 5.2. LC–MS/MS: The Benchmark with Recognized Constraints

Liquid chromatography coupled with tandem mass spectrometry (LC–MS/MS) is generally viewed as the reference method for measuring melatonin because it offers very high sensitivity, strong specificity, and reliable multiplexing. It can detect melatonin together with hormones such as cortisol, cortisone, and serotonin metabolites, allowing for a broad circadian assessment. Even so, several practical issues make routine clinical use difficult. The instruments are expensive to purchase and maintain, and the workflow requires trained personnel for both sample preparation and data processing. Trace-level measurements also increase the risk of carry-over, which means that careful wash cycles and blank injections are necessary. Results can differ between laboratories because of variations in calibrators, chromatographic conditions, and extraction procedures. Harmonization initiatives, such as those reported by (Lanfermeijer et al., 2024 [26]; Jensen et al., 2014 [25]) underline the need for standardized internal standards and common quality controls. In addition, melatonin’s chemical properties—especially its lipophilicity and light sensitivity—require careful handling before analysis. Despite these challenges, LC–MS/MS remains essential in pharmacokinetic studies, in clinical validation of biomarkers, and in multimarker circadian research because of its accuracy and reproducibility [24,27]. While both LC-MS or alternative (e.g., ELISA) methods can be used with mostly similar performance, there are difference in certain aspects of bioanalytical methodology performance and challenges to be taken into account, such as photodegradation of melatonin (20–50% loss) and mass spectrometric matrix effects (internal standard can mitigate this type of issue). The selection of a bioanalytical method for melatonin must be justified by its performance characteristics relative to the intended matrix and study goals. Sensitivity and selectivity LC-MS/MS techniques are unmatched sensitivity, with LODs reaching below 1 pg/mL. Selectivity is superior due highly specific to mass-to-charge ratios and compound-specific fragmentation, whereas spectrophotometric and immunoassay methods are prone to spectral interference and antibody cross-reactivity with precursors like tryptophan or serotonin. Mass spectrometric methods typically report high precision (RSD < 8.3%) and excellent accuracy when using isotopically labeled internal standards to correct for matrix effects. In contrast, ELISA kits often show overestimation bias in saliva and lack the internal standards necessary for absolute quantification in complex samples. Sample preparation methods for LC-MS techniques can range from minimal purification to complex multi-step workflows (solid-phase extraction, liquid–liquid extraction). While LC-MS/MS is the benchmark reference technique, its wider adoption is still limited by high costs for equipment, maintenance and qualified personnel (Table 2).

### 5.3. Hybrid and Emerging Approaches

Recent developments in microextraction, molecularly imprinted polymers (MIPs), and biosensors are bridging the gap between affordability and analytical rigor. For example, MIP–DPX extraction combined with LC–MS/MS (Machado et al., 2025) [7] improves matrix selectivity in lipid-rich samples like breast milk, while reducing solvent use and processing time. In a similar way, nanostructured optical and electrochemical biosensors offer promising options for real-time, point-of-care monitoring of melatonin. These systems use materials such as graphene, gold nanoparticles, or quantum dots to reach picomolar sensitivity without relying on large instruments. Although still under development, these platforms represent a paradigm shift toward personalized circadian diagnostics, where melatonin levels could be monitored continuously through wearable sensors [24].

Advances in analytical techniques and technology have shifted the clinical decision-making from trusting and using lower selectivity and accuracy techniques and methods, such as spectrophotometry, colometry, and even ELISA and immunoassays to more selective and sensitive methods such as LC-MS/MS, partly due to the advances in LC-MS technology over the past two decades (improved sensitivity and robustness) but also due to increasingly lower costs and wider availability of LC-MS analysis. Even if only considering older techniques such as ELISA or immunoassays, recent developments and improvements in these techniques have increased reliability of results offered by these methods. As such, if previously results would sometimes be confirmed via LC-MS analysis on a case-by-case basis (if the technique was available at all) current trends emphasis direct analysis using LC-MS. No single analytical method offers universal superiority. For this reason, the choice of method should be guided by the research question, the type of biological matrix, the required detection range, and the available resources. Consistent procedures for pre-analytical handling, calibration materials, and reporting standards are essential to ensure that results are comparable and reproducible across studies and laboratories [26,28,29]. A diagnostic algorithm for method selection is listed in the table below (Table 3).

### 5.4. Future Perspectives

Advances in nanomaterials, microfluidics, and machine learning are expected to transform melatonin quantification into a more automated, portable, and personalized process. Integrating biosensing technologies with mobile health platforms may soon enable continuous circadian hormone tracking outside laboratory environments. However, for these innovations to become clinically viable, large-scale validation studies, inter-manufacturer standardization, and regulatory harmonization will be necessary [5].

### 5.5. Normalization and Circadian Protocols

To ensure the clinical utility of melatonin measurements, data for urinary aMT6s (creatinine-normalized) is required to account for variations in urine dilution. Determination of urinary aMT6s outperforms salivary melatonin, however, it presents a very high bias of over 25% and thus low confidence.

Furthermore, for studies identifying Dim Light Melatonin Onset (DLMO), sampling protocols must be strictly standardized, often requiring measures to prevent the rapid photodegradation that can reduce measurable concentrations by 20–40%. DLMO sampling frequency (e.g., every 30–60 min) and strict light control (<10 lux) are mandatory to avoid melatonin degradation, confirmed by a >3 pg/mL rise in concentration under controlled conditions, as reported by research groups focusing on this type of determination [2,3]. This controlled sample handling approach is particularly critical in translucent matrices like saliva compared to more “shielded” matrices like hair.

## 6. Conclusions

Analytical techniques for melatonin quantification have evolved substantially over the past decade, offering a spectrum of tools that balance accuracy, cost, and feasibility. Each method carries unique advantages and constraints, and their appropriateness depends largely on the study objectives, biological matrix, and available resources. Accurate melatonin quantification requires more than technical sensitivity—it demands methodological rigor across every step, from sample collection and processing to data interpretation and reporting. By emphasizing standardization, cross-validation, and integration of new technologies, this review supports the development of reliable, sustainable, and clinically meaningful approaches to melatonin biomonitoring.

## Figures and Tables

**Table 1:** Comparison of analytical methods for melatonin quantification across biological matrices.

No	Study (Author, Year)	Method	Biological Matrix	LOD	Linear Range	Cost/ Analysis	Complexity	Multiplexing	Analysis Time	Strengths & Limitations
1.	Khachornsakkul et al., 2023 [9]	dTAD + DLLME	Saliva, urine, serum	2.5 pg/mL	15–45 pg/mL	Low	Medium	No	15–20 min	Low-cost and portable; visual readout introduces subjectivity—digital enhancement recommended.
2.	Dil et al., 2021 [10]	DSPME + UV–Vis	Serum, urine	670 pg/mL	2–500 ng/mL	Low	Medium	No	30–45 min	Simple and green; lacks selectivity of MS—susceptible to spectral interferences.
3.	Martinez Moral & Kannan, 2025 [11]	UPLC–MS/MS	Urine	13 pg/mL	0.02–750 ng/mL	High	High	Yes	60–90 min	Ultra-sensitive; robust multiplexing; requires enzymatic hydrolysis and expensive setup.
4.	Machado et al., 2025 [7]	MIP–DPX + LC–MS/MS	Breast milk	10 pg/mL	Not specified	High	High	Partial	~60 min	Matrix-specific; good selectivity; lipid content complicates extraction.
5.	Yokokawa et al., 2024 [12]	LC–MS/MS	Plasma	0.487 pg/mL	Sub-pg range	High	High	Yes	90 min	Excellent sensitivity; isotopic standards crucial; complex workflow.
6.	Magliocco et al., 2021 [13]	LC–MS/MS	Urine	100 pg/mL	Wide	High	High	No	45 min	Fast method omits hydrolysis, but misses conjugated metabolites.
7.	Zhu et al., 2022 [14]	LC–MS/MS (hair)	Scalp hair	0.1 pg/mg	N/A	High	High	No	2–3 h	Long-term retrospective profiling; slow and labor-intensive.
8.	Eugster et al., 2022 [15]	LC–MS/MS	Plasma	Not specified	Not specified	High	High	Yes	90 min	Broad tryptophan metabolite panel; LOD not reported.
9.	Kaleta et al., 2025 [16]	UHPLC–MS/MS	Plasma	Not specified	Not specified	High	High	Yes	60–75 min	Efficient profiling of 7 key metabolites; minimal sample prep required.
10.	Xu et al., 2023 [17]	UPLC–MS/MS	Urine	50 pg/mL	Wide	High	High	Yes	60–90 min	Clinical application in occupational health; requires enzymatic step.
11.	Duhan et al., 2024 [8]	Optical biosensors	Saliva, Urine, plasma, CSF	<0.232 pg/mL	N/A	Medium	Medium	Potential	10–30 min	Innovative POC-compatible tools; clinical validation still limited.
12.	Oliveira et al., 2022 [20]	ELISA	Cell culture media	Not reported	N/A	Low	Low	No	2–3 h	Easy and accessible; no absolute quantification; suitable for exploratory in vitro studies.
13.	Rzepka-Migut & Paprocka, 2020 [5]	ELISA, RIA, HPLC, LC–MS/MS	Saliva, plasma, urine	<1–100 pg/mL	5–1000 pg/mL	Low ELISA/ High LC-MS/MS	Varies	No	2–6+ h	Comprehensive method overview; variable standardization; emphasizes pre-analytical challenges.
14.	Kaleta M. et al., 2025 [16]	UHPLC–MS/MS	Plasma	Not specified	0.01–100 ng/mL;	High	High	Yes	<10 min	Sensible and specific, but high cost and high complexity.
15.	Sofu U et al., 2025 [19]	Polymer-based electrochemical sensor	Plasma, urine	5668 pg/mL (DPV)/12,033 pg/mL (SWV)	24.4 nM (DPV)/51.8 nM (SWV)	Low	Moderate	No	1–3 min	Fast, low cost, but higher LOD than for LC-MS/MS.

Legend: Cost/Analysis: Low: < €10/test, Medium: €10–50/test, High: > €50/test. Complexity: Low: simple assays with minimal sample preparation, Medium: basic extraction or processing steps, High: advanced equipment, trained personnel, and extensive validation. LOD—limit of detection; N/A—not applicable.

**Table 2:** Comparison of typical performance for LC-MS and ELISA methods.

Method	LOD (pg/mL)	Selectivity/Specificity	Bias (%)	CV (%) (Intra/Inter Run)	Sample Prep Time (Min)	Known Issues
LC-MS	<1	>99%	±5	<5/<10	30–60	Carryover
ELISA	5–10	80–90%	+15	<15/<20	<10	Cross-react

**Table 3:** Diagnostic algorithm for method selection.

Scheme 12	Recommended Method	Rationale
Clinical pharmacokinetic trials	LC–MS/MS or UHPLC–MS/MS [12,13]	Gold standard for sensitivity, specificity, and multiplex biomarker quantification
Hospital or research laboratories with limited resources	Validated ELISA (cross-checked with LC–MS/MS) [19]	Cost-effective for screening and circadian rhythm assessment
Field or epidemiological studies	Colorimetric/spectrophotometric (dTADs, DSPME–UV–Vis) [9]	Portable, affordable, suited for high-throughput
Breast milk, saliva, or complex lipid matrices	MIP–DPX LC–MS/MS [11]	Enhanced selectivity and reduced matrix interference
Point-of-care or wearable diagnostics	Optical/electrochemical biosensors [18]	Real-time, minimally invasive, patient-centered monitoring

## Data Availability

No new data were created or analyzed in this study. Data sharing is not applicable to this article.
